# Supplementary material for: The effect of deep brain stimulation on cortico-subcortical networks in Parkinson’s disease patients with freezing of gait: Exhaustive exploration of a basic model
Source: Netw Neurosci. 2024 Oct 1;8(3):926–45. doi: 10.1162/netn_a_00376 (PMC11424038; doi:10.1162/netn_a_00376)
Supplement: Supplementary file 1 [file netn-8-3-926-s001.pdf]

Journal: NETWORK NEUROSCIENCE

RESEARCH

Supplement to: The effect of deep brain stimulation on cortico-subcortical networks in Parkinson's disease patients with freezing of gait: Exhaustive exploration of a basic model

Mariia Popova[1], Arnaud Messé[1], Alessandro Gulberti[2],

Christian Gerloff[2], Monika Pötter-Nerger[2] and Claus C Hilgetag[1]

[1] Institute of Computational Neuroscience, Hamburg Center of Neuroscience, University Medical Center Hamburg-Eppendorf, Hamburg University, Hamburg, Germany

[2] Department of Neurology, Hamburg Center of Neuroscience, University Medical Center Hamburg-Eppendorf, Hamburg University, Hamburg, Germany

This document contains supplementary figures and a set of null tests to the article entitled "The effect of deep brain stimulation on cortico-subcortical networks in Parkinson's disease patients with freezing of gait: Exhaustive exploration of a basic model".

Link to the repository: https://github.com/mariiapopova/FOG-SER

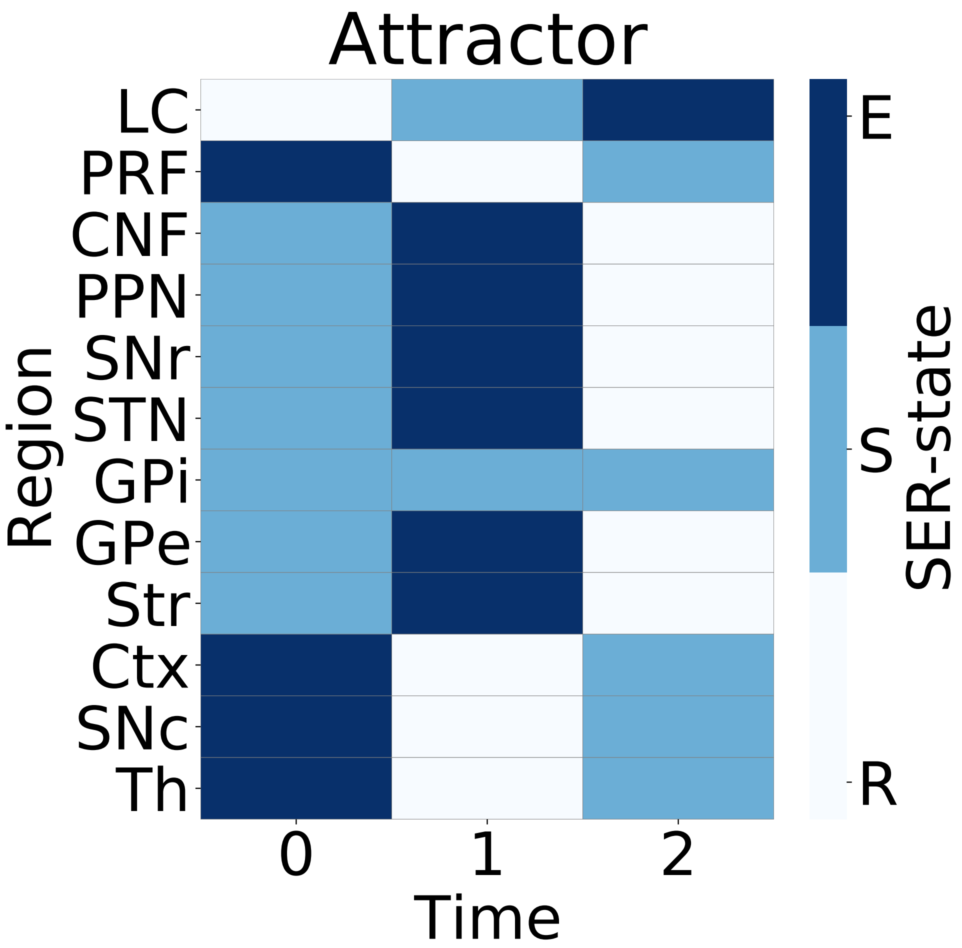

Figure S1. Limit cycle with the largest basin of attraction for the STN DBS configuration. Y-axis shows different regions of the FoG network, while X-axis shows time steps. States of the SER model are color-coded.

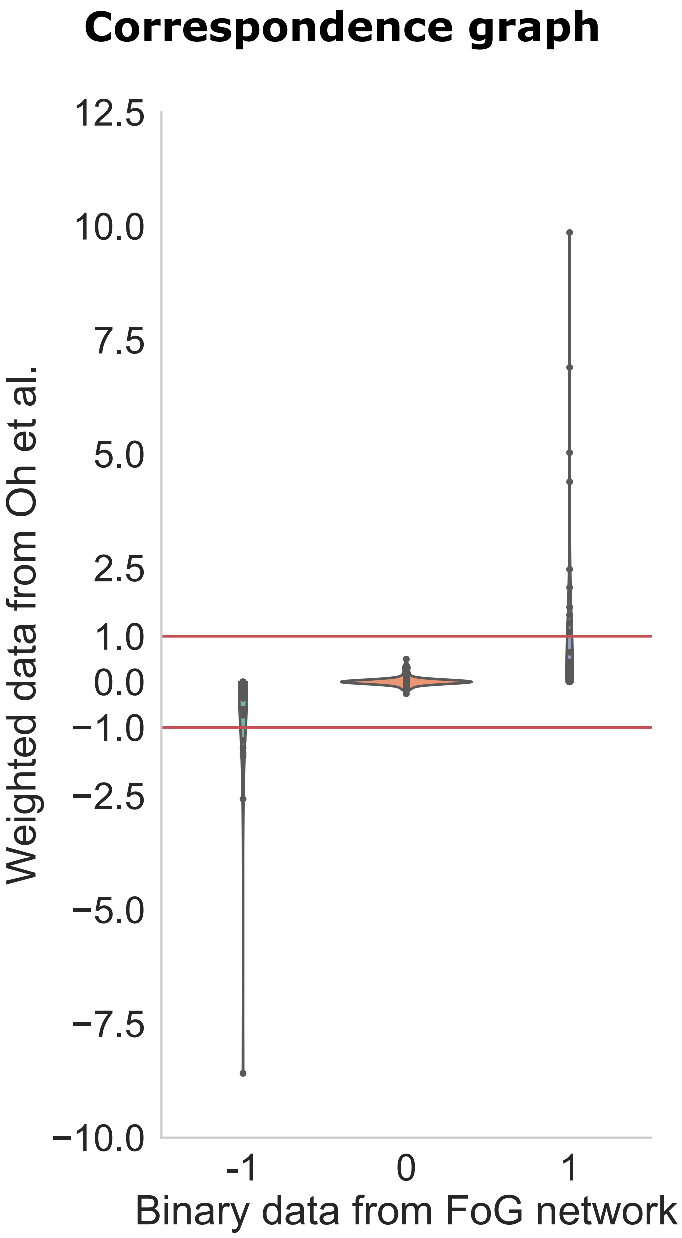

Figure S2. Connectome comparison.

The edge weights from (Oh et al., 2014) (Y-axis) are grouped along the binary weights of the corresponding edges used in healthy FoG network configuration (X-axis). The red lines show 1 and -1 weights borders.

NULL TESTS

A null test for the healthy network configuration

To demonstrate that network dynamics change significantly depending on the choice of the topology, we randomly shuffle the network, preserving its outdegree distribution. For this, we used the network randomization method implemented in the Brain Connectivity toolbox (Rubinov & Sporns, 2010). The dynamical patterns of the new network are summarized in Table 1. We also depict them in form of coactivation matrix (Figure S3). No limit cycles with a period other than 3 is found for the new network configuration.

Table 1. A summary of dynamical landscapes for the random network configuration

	**Healthy**
Number of fixed points	399893 (75%)
Number of period-3 limit cycles	131548 (25%)
Number of unique limit cycles	52
Largest limit cycle in a limit cycle space	25%

Note. The numbers of fixed points and period-3 limit cycles are also given in percentages with respect to the total number of initial conditions ($3^{12} = 531.441$). The last row shows the percentage fraction of the limit cycle with the largest basin of attraction in a limit cycle space.

Indeed, the dynamical pattern differs for the randomized network. There are more limit cycles, as well as more unique limit cycles compared to the healthy network configuration. The largest limit cycle takes up more space in terms of its basin of attraction. Also, the coactivation matrix appears to be more modular in comparison to the network in the healthy configuration.

A null test for the PD network configuration

To demonstrate possible changes observed in the network due to the edge removal, we create two new network configurations: N1 and N2. Similarly to the PD configuration, these configurations are obtained from the healthy one by setting the weights of edges originating from a certain node to 0. In the case of

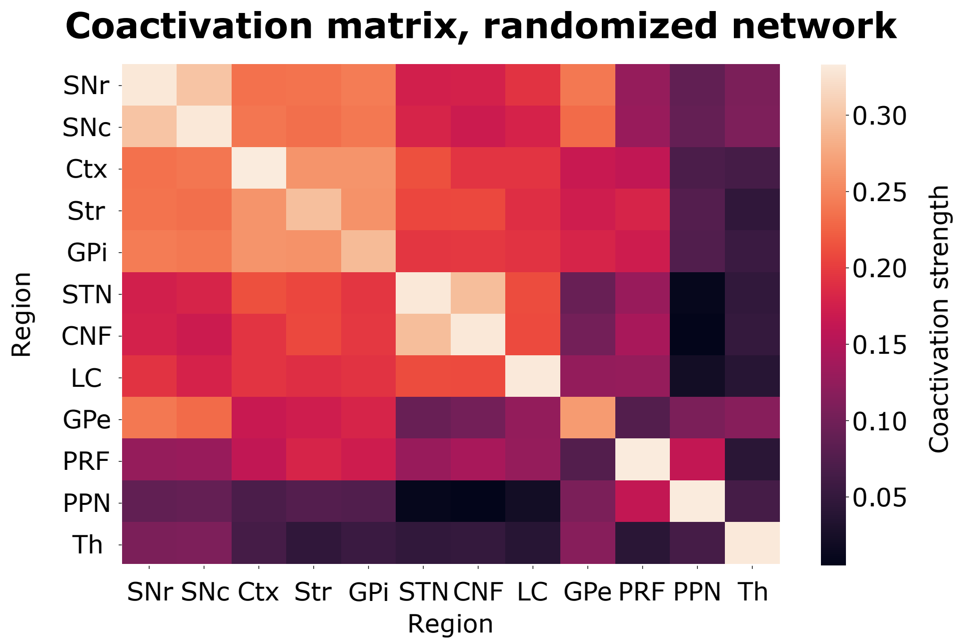

Figure S3. Coactivation matrix of the network used for a null test.

The coactivation pattern differs from the one for the healthy network configuration.

the N1 configuration, we set the edges coming out of the Str to 0, while for the N2, the edges coming out from the Th are set to 0. The dynamical patterns of the new networks are summarized in Table 2. We also depict them in the form of Venn diagrams (Figures S4, S5). No limit cycles with a period other than 3 is found for the new network configurations.

There is a change in dynamical landscapes for N1 and N2 configurations compared to the PD configuration. In the case of the N1 configuration, there is an additional limit cycle that is similar to the healthy and N1 configuration (Figure S4). This leads to a further decrease in the fraction of the basin of attraction of the unique healthy attractors. In the case of the N2 configuration, there are fewer emerging unique N2 attractors when compared to the PD configuration (Figure S5). The fraction of the basin of attraction related to them is also significantly smaller. In addition, Table 2 shows that the overall number of limit cycles is decreasing in the case of the N2 configuration.

While the dynamical landscape for the N1 configuration is more similar to the PD configuration, the dynamical landscape for the N2 configuration drastically differs. One explanation could be that when creating the N2 configuration, we delete edges coming out from the Th. These edges are excitatory and

Table 2. A summary of dynamical landscapes for the new network configurations

	**N1**	**N2**
Number of fixed points	379440 (71%)	462750 (87%)
Number of period-3 limit cycles	152001 (29%)	68691 (13%)
Number of unique limit cycles	57	25
Largest limit cycle in a limit cycle space	32%	17%

Note. The numbers of fixed points and period-3 limit cycles are also given in percentages with respect to the total number of initial conditions ($3^{12} = 531.441$). The last row shows the percentage fraction of the limit cycle with the largest basin of attraction in a limit cycle space.

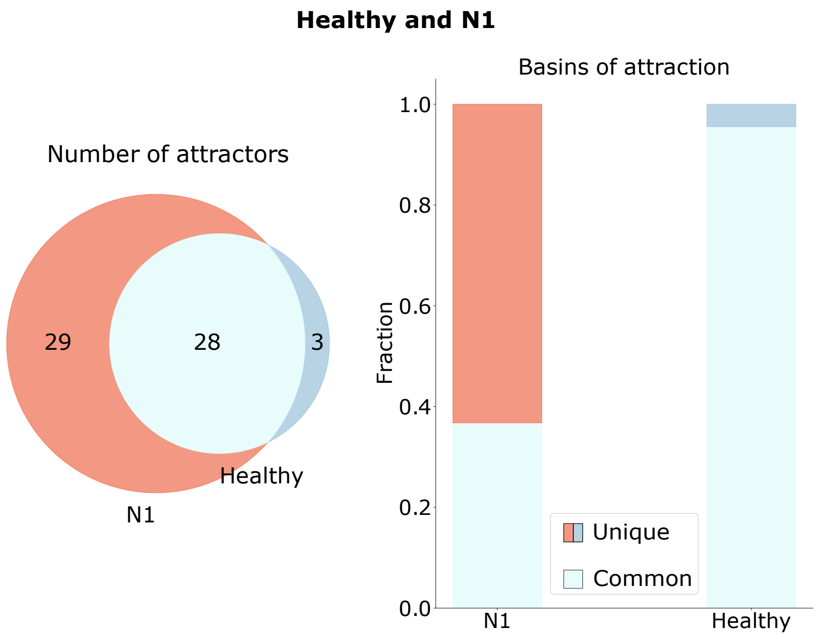

Figure S4. Healthy and N1 limit cycle spaces.

Limit cycles unique to the healthy and N1 configurations are shown in blue and red, respectively. Limit cycles, which are similar in the healthy and N1 configurations, are shown in light blue. The numbers inside the circles are the numbers of the limit cycles of an aforementioned type. On the right side, the basins of attractions are compared across configurations, with the fraction of the basin size to the number of limit cycles depicted along the Y-axis.

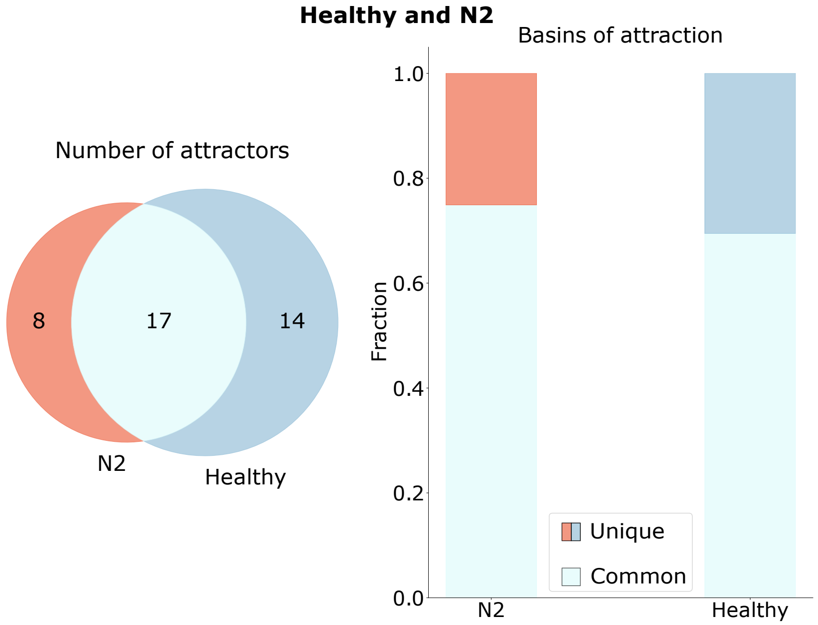

Figure S5. Healthy and N2 limit cycle spaces.

Limit cycles unique to the healthy and N2 configurations are shown in blue and red, respectively. Limit cycles, which are similar in the healthy and N2 configurations, are shown in light blue. The numbers inside the circles are the numbers of the limit cycles of an aforementioned type. On the right side, the basins of attractions are compared across configurations, with the fraction of the basin size to the number of limit cycles depicted along the Y-axis.

form topological loops. Following this deletion, the propagation of activity across the network is hindered. On the contrary, the N1 configuration leads to the deletion of the inhibitory nodes like the original PD configuration.

A null test for the STN+SNr DBS network configuration

To demonstrate that the degree of increase in heterogeneity of dynamical patterns is specific to the choice of the stimulation target sites, we create an STN+Str DBS network configuration. The STN+Str DBS configuration was created from the PD by setting the weights of all the edges originating from both the STN node and the Str node to 0.

In Figure S6, the limit cycle spaces of the healthy, the PD, and the STN+Str DBS configurations are compared. In contrast to the STN+SNr DBS, one can see that less new attractors appear that are not present in the attractor space of the PD or healthy configurations (in yellow). There are 12 new limit cycles. However, the whole system does not move into the new dynamical space. This is due to the pattern

that could be seen when comparing basins of attraction (right side of Figure S6). The basin of attraction of unique attractors of the STN+Str DBS configuration (in yellow, 11% of all the limit cycle space for the STN+Str DBS configuration) is much smaller than the one of limit cycles, which are similar in the STN+Str DBS and PD configurations. From an additional analysis, we find that contrary to the STN+SNr DBS configuration, the network in the STN+Str DBS configuration does not move away from the limit cycle with the largest basin of attraction in the PD configuration.

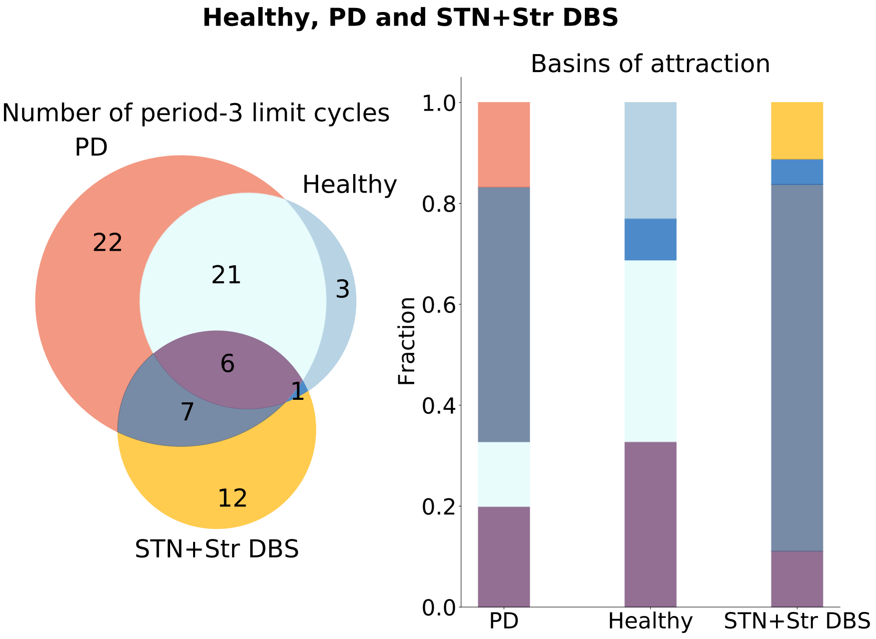

Figure S6. Healthy, PD, and STN+Str DBS limit cycle spaces.

Limit cycles unique to the healthy, PD, and STN+Str DBS configurations are shown in blue, red, and yellow, respectively. Limit cycles, which are similar in the healthy and PD configurations, are shown in light blue. Limit cycles, which are similar in the STN+Str DBS and PD configurations, are shown in gray. Limit cycles, similar in the STN+Str DBS and healthy configurations, are shown in dark blue. Limit cycles, which are similar across all configurations, are shown in purple. The numbers inside the circles are the numbers of the limit cycles of an aforementioned type. On the right side, the basins of attractions are compared across configurations, with the fraction of the basin size to the number of limit cycles depicted along the Y-axis. The colors correspond to the colors on the left panel.

The same pattern is noticeable when comparing distances between the coactivation matrices (Figure S7). Indeed, the STN+Str DBS coactivation matrix is less similar to the healthy state coactivation matrix relative to the PD configuration. This is different from the dynamical behavior in the STN+SNr configuration.

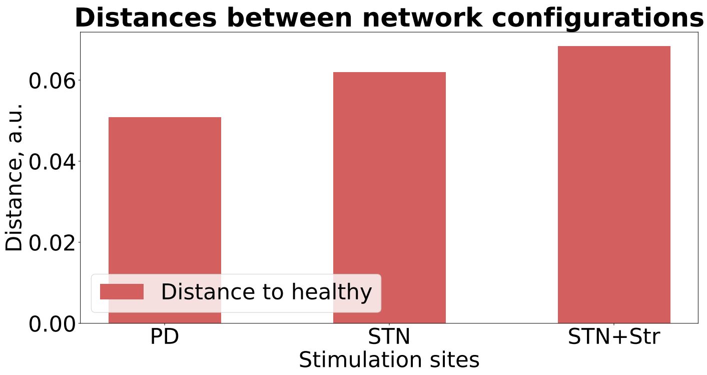

Figure S7. 1-norm distances between the coactivation matrices of the PD, STN DBS and STN+Str DBS to the healthy state.

The STN+Str DBS coactivation matrix is less similar to the healthy state coactivation matrix compared to the STN DBS relative to the PD configuration.

EXCITABILITY OF NODES

In this section, we study the relationship between the node effective indegree (Figure S8 A), a sum of excitatory and inhibitory projections to each node, and its excitability (Figure S8 B, C).

Effective indegree of the nodes varies depending on the network configuration due to the topological changes needed to form the configurations. To simulate the PD configuration, we delete edges coming out of the SNc, which leads to an indegree of nodes that is higher on average than those in the healthy state. This is due to projections outgoing from the SNc being predominantly inhibitory. The opposite trend is seen for the STN DBS configuration. In this case, we delete excitatory projections; thus, the effective indegree of the nodes decreases (yellow line in Figure S8 A).

Additionally, effective indegree varies from node to node as a consequence of the chosen network topology. For example, for the Str node in all the different topological configurations, it is below 0. However, it does not prevent the Str node from entering the state E under the SER model. For the node to enter the state E, we need to consider only the indegree produced by the neighbors of that node excited on the previous time step. That is why the effective indegree under the 0 threshold does not preclude the node from

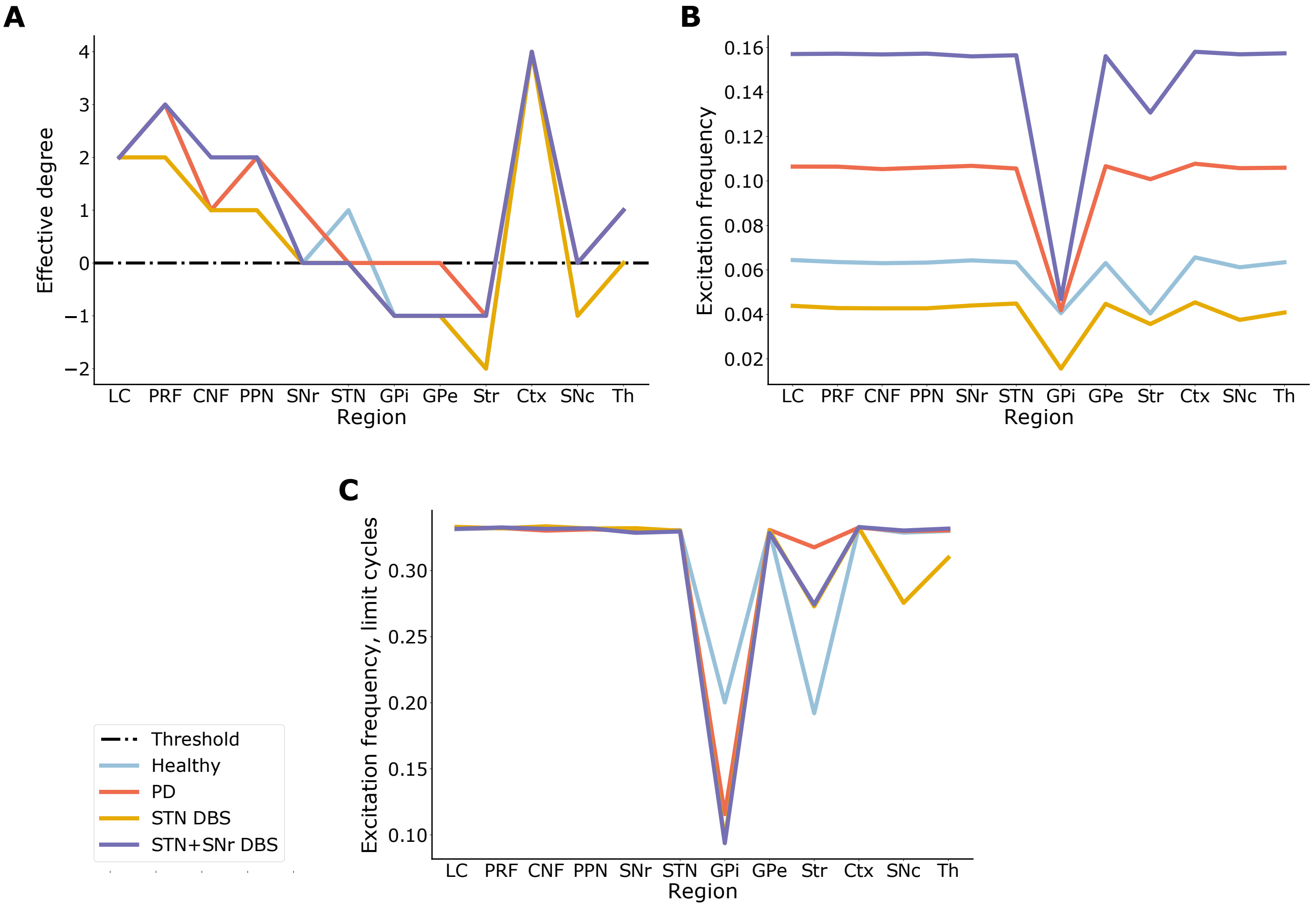

Figure S8. Relationship between effective indegree of the nodes and their excitability. Blue corresponds to the healthy, red to the PD, yellow to the STN DBS, and purple to the STN+SNr DBS network configurations. (A) Effective indegree of the nodes. Black dotted line depicts excitability threshold 0 in terms of effective indegree. (B) Excitation frequency of the nodes. (C) Excitation frequency of the nodes when considering only limit cycles.

entering the E state but makes it less probable. This trend could be observed in Figure S8 B, C. Here, the excitation frequency was calculated as a frequency of all the E state occurrences across all initial condition space over the simulation time for a node of choice. Indeed, the excitation frequency (Figure S8 B) varies depending on the network configuration. It is slightly lower for the network configuration obtained via the removal of excitatory edges (STN-DBS). The trend is the opposite for the STN+SNr DBS. The effects of the low effective indegree are also seen as dips for the GPi and Str. Figure S8 C shows the excitation frequencies when considering only the limit cycles. Here, the effects of the network configurations are less evident due to the exclusion of the fixed points. However, the dips due to low indegree are more prominent

for the GPi and the Str. It must be noted that there is no reduction in terms of excitation frequency for the GPe, even though its effective indegree is below 0. This suggests that effective indegree does not fully explain the excitation patterns of the network while contributing to the observed effects.

REFERENCES

Oh, S. W., Harris, J. A., Ng, L., Winslow, B., Cain, N., Mihalas, S., … Zeng, H. (2014). A mesoscale connectome of the mouse brain. *Nature*, *508*(7495), 207–214. doi: 10.1038/nature13186

Rubinov, M., & Sporns, O. (2010). Complex network measures of brain connectivity: uses and interpretations. *Neuroimage*, *52*(3), 1059–1069.
